# Selenium-binding protein 1 transcriptionally activates p21 expression via p53-independent mechanism and its frequent reduction associates with poor prognosis in bladder cancer

**DOI:** 10.1186/s12967-020-02211-4

**Published:** 2020-01-09

**Authors:** Yulei Wang, Wenzhen Zhu, Xiaoqing Chen, Guangnan Wei, Guosong Jiang, Guochun Zhang

**Affiliations:** 1 Cancer Center, Guangdong Provincial People’s Hospital and Guangdong Academy of Medical Sciences, 106 Zhongshan Er Road, Guangzhou, 510080 China; 2 School of Medicine, South China University of Technology, Guangzhou, 510641 China; 3 Department of Urology, Union Hospital, Tongji Medical College, Huazhong University of Science and Technology, Wuhan, 430022 China

**Keywords:** Selenium-binding protein 1 (SELENBP1), p21, p53, c-Jun, STAT1, Methylation, Bladder cancer

## Abstract

**Background:**

Recent studies have shown that selenium-binding protein 1 (SELENBP1) is significantly down-regulated in a variety of solid tumors. Nevertheless, the clinical relevance of SELENBP1 in human bladder cancer has not been described in any detail, and the molecular mechanism underlying its inhibitory role in cancer cell growth is largely unknown.

**Methods:**

SELENBP1 expression levels in tumor tissues and adjacent normal tissues were evaluated using immunoblotting assay. The association of *SELENBP1* expression, clinicopathological features, and clinical outcome was determined using publicly available dataset from The Cancer Genome Atlas bladder cancer (TCGA-BLCA) cohort. DNA methylation in *SELENBP1* gene was assessed using online MEXPRESS tool. We generated stable SELENBP1-overexpression and their corresponding control cell lines to determine its potential effect on cell cycle and transcriptional activity of p21 by using flow cytometry and luciferase reporter assay, respectively. The dominant-negative mutant constructs, TAM67 and STAT1 Y701F, were employed to define the roles of c-Jun and STAT1 in the regulation of p21 protein.

**Results:**

Here, we report that the reduction of SELENBP1 is a frequent event and significantly correlates with tumor progression as well as unfavorable prognosis in human bladder cancer. By utilizing TCGA-BLCA cohort, DNA hypermethylation, especially in gene body, is shown to be likely to account for the reduction of *SELENBP1* expression. However, an apparent paradox is observed in its 3′-UTR region, in which DNA methylation is positively related to *SELENBP1* expression. More importantly, we verify the growth inhibitory role for SELENBP1 in human bladder cancer, and further report a novel function for SELENBP1 in transcriptionally modulating p21 expression through a p53-independent mechanism. Instead, ectopic expression of SELENBP1 pronouncedly attenuates the phosphorylation of c-Jun and STAT1, both of which are indispensable for SELENBP1-mediated transcriptional induction of p21, thereby resulting in the G_0_/G_1_ phase cell cycle arrest in bladder cancer cell.

**Conclusions:**

Taken together, our findings provide clinical and molecular insights into improved understanding of the tumor suppressive role for SELENBP1 in human bladder cancer, suggesting that SELENBP1 could potentially be utilized as a prognostic biomarker as well as a therapeutic target in future cancer therapy.

## Background

Bladder cancer is one of the most common urological malignancies, and is the leading cause of deaths in patients with urinary tract disease. The incidence of bladder cancer has steadily increased worldwide in recent decades [1]. According to the report from American Cancer Society in 2018, there are an estimated 81,190 of new cases diagnosed with bladder cancer and approximately 17,240 of bladder cancer-related deaths in the United States [2]. For non-muscle-invasive and muscle-invasive bladder cancer, grade and depth of tumor invasion are the most important prognostic factors, respectively [3]; the 5-year overall survival rate is approximately 8.1% among patients with distant metastasis [4]. Therefore, the identification of novel therapeutic targets or predictive biomarkers, and in-depth elucidation of the underlying molecular mechanisms are of tremendous importance for reducing the mortality of this disease.

The human selenium-binding protein 1 (*SELENBP1*) gene, located on chromosome 1 at q21-22, encodes a highly-conserved member of the selenium-binding protein family [5], and is the homologue of the mouse *SP56* gene that has been originally reported as a 56 kDa mouse protein with the ability to bind ^75^selenium stably [6, 7]. The human SELENBP1 protein is ubiquitously expressed in various tissue types, especially higher in heart, lung, liver and kidney [5]. Since the initial report identifying SELENBP1 as a tumor-associated protein in prostate cancer [8], the reduced expression or even lost of SELENBP1 has been consistently observed in a variety of solid tumors as compared to corresponding normal tissues, including those of the skin [9], lung [10], esophagus [11], stomach [12–14], colon [15, 16], liver [17], breast [18] and ovary [19]. Thereafter, accumulating evidence has convincingly demonstrated that reduced expression of SELENBP1 is an independent predictive of poor clinical outcome in multiple malignant diseases [13–18, 20–22]. Furthermore, an increasing number of in vitro and in vivo studies has also consistently shown that increasing the levels of SELENBP1 significantly suppresses the malignant characteristics of cancer cells, leading scientific community to the consensus that SELENBP1 may act as a putative tumor suppressor involved in the regulation of cell proliferation, senescence, epithelial–mesenchymal transition, migration and apoptosis [23]. However, the clinical significance of SELENBP1 in human bladder cancer has not yet been characterized in any detail. Additionally, the molecular mechanisms underlying the tumor-suppressive role for SELENBP1 in cancer cells are still largely undefined.

Here we show that SELENBP1 is significantly down-regulated in human bladder cancer tissues and cell lines, and its frequent reduction is further associated with tumor progression as well as poor clinical outcome among patients with bladder cancer. In addition, the reduced expression of *SELENBP1* is inversely associated with DNA hypermethylation in its promoter region, but more significantly correlated negatively with DNA methylation in gene body. Importantly, an apparent paradox is observed in 3′-UTR region, in which DNA methylation is positively related to *SELENBP1* expression. Furthermore, we verify the growth inhibitory role for SELENBP1 in human bladder cancer, and report a novel function for SELENBP1 in transcriptionally modulating p21 expression through a p53-independent mechanism. Instead, ectopic expression of SELENBP1 pronouncedly attenuates the phosphorylation of c-Jun and STAT1, both of which are indispensable for SELENBP1-mediated transcriptional induction of p21, thereby resulting in the G_0_/G_1_ phase cell cycle arrest in bladder cancer cell.

## Methods and materials

### Cell lines, cell culture and plasmids

Human bladder cancer cell lines (UMUC3, RT4, RT112, TCCSUP, J82 and 5637) used in this study were described previously [24, 25]. Human bladder cancer T24 and its metastatic derivative T24T cell lines [26], and human colon cancer wild-type HCT116 (HCT116 WT), p21 knockout (HCT116 p21^−/−^) and p53 knockout (HCT116 p53^−/−^) cell lines [27] were described in our prior studies, and were cultured in corresponding medium supplemented with 10% heat-inactivated fetal bovine serum, 2 μM l-glutamine and 25 μg/mL gentamycin at 37 °C in a humidified atmosphere of 5% CO_2_. As previously described [28, 29], UMUC3 and T24T cell lines were authenticated by Genetica DNA Laboratories (Burlington, NC, USA). The constructs encoding human HA-tagged SELENBP1 (HA-SELENBP1) [30] or dominant-negative form of c-Jun (TAM67) [31] were previously described. The plasmid encoding dominant-negative STAT1 (DN-STAT1 Y701F) and its control empty vector (pEGFP-C1) were gifts from Alan Perantoni (Addgene plasmid #12302) [32]. Full-length (2.4 Kb) and truncated-length (1.8 Kb, 1.3 Kb and 200 bp) of p21 promoter-driven luciferase reporter plasmids were generously provided by Dr. Jennifer A. Pietenpol at Vanderbilt Ingram Comprehensive Cancer Center, Vanderbilt University School of Medicine (Nashville, TN) [33].

### Human bladder cancer tissue specimens

Twelve pairs of primary invasive bladder cancer specimens and their paired adjacent normal bladder tissues were obtained from patients who received radical cystectomy from 2012 to 2013 at the Department of Urology of the Union Hospital of Tongji Medical College (Wuhan, China) [34]. After surgical procedure, all of specimens were immediately collected and stored in liquid nitrogen, and the pathological diagnoses were further confirmed by a certified clinical pathologist according to the 2004 World Health Organization Consensus Classification and Staging System for bladder neoplasms [35]. Before collecting these specimens, ethical approval has been obtained from the Medical Ethics Committee of China, and all patients have given their informed consent for translational research.

### Transfection and luciferase reporter assay

All of stable or transient transfections were performed using PolyJet™ DNA in vitro transfection reagent (SignaGen Laboratories, Rockville, MD) according to manufacturer’s instructions. Stable transfectants were selected with puromycin, hygromycin B or G418 for 3–4 weeks [26, 27], and surviving cells from the antibiotics selection were pooled as stable mass transfectants, with at least two passages prior to utilization for further experiments. For the determination of p21 promoter-mediated luciferase activity, cells were transfected with the related luciferase reporter in combination with the pRL-TK vector (Promega, Madison, WI). After 24 h of transfection, luciferase activity was determined using a dual-luciferase reporter assay system kit (Promega, Madison, WI) as described previously [36, 37]. The results were normalized by internal TK signal, and expressed as mean ± standard deviation from at least three independent experiments.

### Immunoblotting assay and antibodies

Whole-cell extracts were prepared with cell lysis buffer (10 mM Tris–HCl (pH 7.4), 1% SDS, and 1 mM Na_3_VO_4_), and were then subjected to immunoblotting analysis as described in our previous studies [24, 27]. Briefly, equal aliquots of cell extracts were resolved by SDS-PAGE, transferred to PVDF membranes (Bio-Rad, Hercules, CA) and probed with primary antibodies against one of the following proteins: SELENBP1 (Medical and Biological Laboratories, Japan), p21, p27, Cyclin D1, CDK4, CDK6, p53, Sp1, JunB, Jun D, c-Jun, phospho-c-Jun at S73, STAT1/3/5/6, phospho-STAT1 at Y701, phospho-STAT3 at Y705, phospho-STAT5 at Y694, phospho-STAT6 at Y641, SOCS1, GAPDH and HA-tag (Cell Signaling Technology, Beverly, MA). Primary-antibody-bound proteins were further incubated with an alkaline-phosphatase-conjugated secondary antibody and detected using an ECF immunoblotting system (Amersham, Piscataway, NJ). Images were acquired using Typhoon FLA 7000 (GE Healthcare, Pittsburgh, PA). The results shown are representative of three independent experiments. The density of bands was quantified relative to that of loading control by using Quantity One software (Life Science, Hercules, CA) [31].

### RT-PCR

Total RNA was extracted using TRIzol reagent (Invitrogen, Grand Island, NY) according to the manufacturer’s instructions. Total RNA (5.0 μg) was used for first-strand cDNA synthesis with oligo (dT) 20 primer by SuperScript First-Strand Synthesis system (Invitrogen). The mRNA levels were determined by semi-quantitative reverse transcription (RT)-PCR, as described previously [38]. These specific primers used in this study were as follows: human *p21* (forward, *5′*-*CCC AGT GGA CAG CGA GCA GC*-*3′*; reverse, *5′*-*ACT GCA GGC TTC CTG TGG GC*-*3′*), and human *GAPDH* (forward, *5′*-*AGA AGG CTG GGG CTC ATT TG*-*3′*; reverse, *5′*-*AGG GGC CAT CCA CAG TCT TC*-*3′*).

### Anchorage-independent growth assay

Anchorage-independent growth assay in soft agar (soft agar assay) was performed as described in our previous study [31, 39]. Briefly, 1 × 10^4^ of indicated cells were re-suspended in 1 mL of 10% FBS basal modified Eagle’s medium (BME) containing 0.33% soft agar and seeded on top of 0.5% agar in 10% FBS BME in each well of 6-well plates. The plates were maintained in a 5% CO_2_ incubator at 37 °C for 2–3 weeks. Colonies with more than 32 cells were scored and presented as mean ± standard deviation from at least three independent experiments.

### Cell-cycle analysis

As previously described [27], the indicated cells were harvested and fixed in 75% ethanol overnight, and were suspended in the staining buffer containing 0.1% Triton X-100, 0.2 mg/mL RNase A, and 50 mg/mL propidium iodide (PI) at 4 °C. DNA content was then determined by a flow cytometry Epics XL flow cytometer (Beckman Coulter Inc., Miami, FL) and the results were analyzed with EXPO32 (v1.2) software.

### Bioinformatics analysis of data mining in TCGA-bladder cancer (TCGA-BLCA) cohort

The TCGA-BLCA clinical data and *SELENBP1* expression data were obtained from UCSC Xena Browser (https://xenabrowser.net; last updated on April 5th 2019) [40]. UCSC Xena browser shows that TCGA has 406 patients with bladder cancer, in which 19 of patients have *SELENBP1* expression data of primary tumor and matched normal bladder tissues. Patients are categorized into low or high *SELENBP1* expression group using cut-off value of *X*-2.33*SD* (*X*, mean; *SD*, standard deviation) calculated from *SELENBP1* expression in the matched normal bladder tissues. These two groups were applied for Kaplan–Meier survival analysis (10-year survival). The correlation between *SELENBP1* expression and clinicopathological parameters in TCGA-BLCA were also examined. Besides, we analyzed the association between *SELENBP1* expression and DNA methylation using the MEXPRESS tool (http://mexpress.be/), a user-friendly tool for the visualization and interpretation of DNA methylation in TCGA data [41].

### Statistical analysis

Statistical analysis was performed using SPSS 19.0 software (SPSS, Chicago, IL). Student’s t-test or Fisher’s exact test was used to determine significant differences. *p* < 0.05 is considered statistically. The results are expressed as the mean ± standard deviation from at least three independent experiments.

## Results

### SELENBP1 is frequently down-regulated in human bladder cancer, and low expression of SELENBP1 predicts poor clinical prognosis

**Fig. 1:**
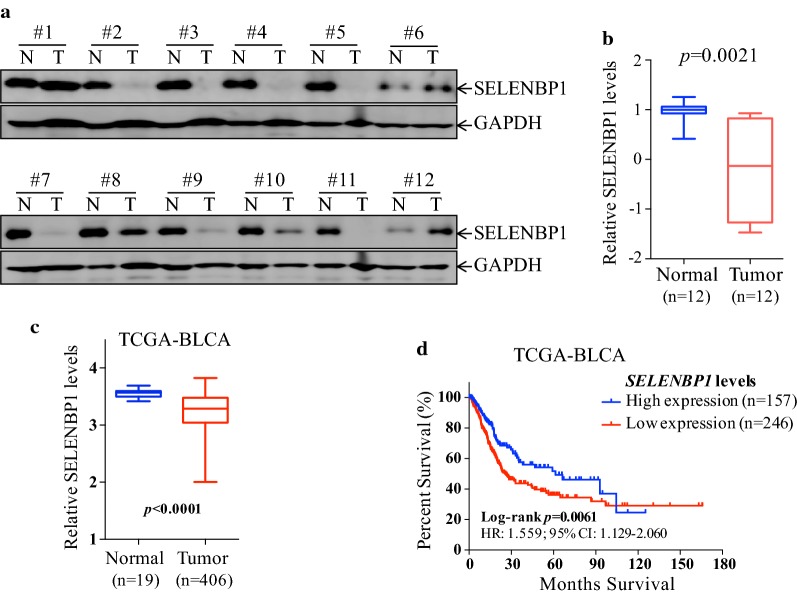
SELENBP1 is frequently down-regulated in human bladder cancer, and low expression of SELENBP1 predicts poor clinical prognosis. **a** Twelve pairs of bladder cancers (T) and adjacent matched normal tissues (N) were extracted and subjected to immunoblotting assay for determining the levels of SELENBP1 protein expression. GAPDH was used as a loading control. **b** Relative intensity of SELENBP1 in **a** was determined using Quantity One software, and normalized to GAPDH. The relative intensity of SELENBP1 in normal tissues was set to 1.0. Paired t test was performed and *p* value was indicated in top panel. **c***SELENBP1* mRNA levels were extracted from TCGA bladder cancer (TCGA-BLCA) cohort, including 19 of normal bladder tissues and 406 of bladder cancer tissues. Non-paired t test was performed and *p* value was indicated in the panel. **d** Kaplan–Meier 10-year survival analysis among patients with high and low *SELENBP1* expression in TCGA-BLCA cohort. The log-rank test was performed and *p* value was indicated in the panel. Hazard ration (HR) and 95% confidence interval (CI) were also calculated

To determine SELENBP1 expression levels in human bladder cancer, we evaluated the protein expression of SELENBP1 in 12 pairs of bladder cancer tissues and matched adjacent normal tissues using immunoblotting assay. As shown in Fig. 1a, b, SELENBP1 protein levels were significantly down-regulated in 9 out of 12 bladder cancers relative to matched adjacent normal tissues (*p *= 0.0021). We further examined *SELENBP1* mRNA levels in The Cancer Genome Atlas bladder cancer (TCGA-BLCA) cohort, including 406 patients with *SELENBP1* expression data, in which 19 of patients have *SELENBP1* expression data of primary tumor and matched normal bladder tissues. Consistently, reduction of *SELENBP1* expression was also frequently observed in a majority of tumor tissues from TCGA-BLCA cohort (*p *< 0.0001; Fig. 1c). Utilizing the gene expression data in matched normal tissues, we created two groups based on *SELENBP1* gene expression and analyzed overall survival (OS) curves of differential *SELENBP1* expression levels. Log-rank test of the OS curves in TCGA-BLCA cohort indicated that bladder cancer patients with low *SELENBP1* expression were remarkably related to worse prognosis than those with high *SELENBP1* expression (Fig. 1d), implying that SELENBP1 might serve as a tumor suppressor in bladder cancer.

### Low expression of SELENBP1 correlates with unfavorable clinicopathological characteristics of bladder cancer patients

Next, we analyzed the relationship between *SELENBP1* expression and clinicopathological features in bladder cancer by using TCGA-BLCA cohort (Table 1), and found that low expression of *SELENBP1* was markedly correlated with non-papillary subtype (Papillary: 52% vs. Non-papillary: 64%; *p *= 0.023), and advanced tumor-node-metastasis (TNM) stage (I/II: 53% vs. III/IV: 64%; *p *= 0.029), and distant metastasis with marginal statistical significance (*p *= 0.057), but not with age, gender, lymphovascular invasion (LVI) or other clinicopathological features. These results strongly suggest that the reduced expression of *SELENBP1* is associated with the aggressive phenotype of bladder cancer.

**Table 1:** The correlation of *SELENBP1* expression with clinicopathological features in TCGA-BLCA cohort (n = 406)

Variables	*SELENBP1* expression		*p*-value
	High (n = 160)	Low (n = 246)	
Age (years)			
< 70	86 (40%)	128 (60%)	0.761
≥ 70	74 (39%)	118 (61%)	
Sex			
Female	44 (42%)	62 (58%)	0.644
Male	116 (39%)	184 (61%)	
Histologic subtype			
Papillary	63 (48%)	69 (52%)	*0.023*^a^
Non-papillary	96 (36%)	173 (64%)	
Missing	1 (20%)	4 (80%)	
Histologic grade			
Low grade	9 (43%)	12 (57%)	0.819
High grade	149 (39%)	233 (61%)	
Missing	2 (67%)	1 (33%)	
Primary tumor			
T1/T2	56 (46%)	66 (54%)	0.090^b^
T3/T4	91 (36%)	160 (64%)	
Missing	13 (39%)	20 (61%)	
Regional lymph node			
Negative	93 (40%)	142 (60%)	0.430^c^
Positive	45 (35%)	84 (65%)	
Missing	22 (52%)	20 (48%)	
Distant metastasis			
M0	76 (39%)	120 (61%)	0.057^d^
M1	1 (9%)	10 (91%)	
Missing	83 (42%)	116 (58%)	
Lymphovascular invasion (LVI)			
Negative	59 (46%)	70 (54%)	0.469^e^
Positive	62 (41%)	89 (59%)	
Missing	39 (31%)	87 (69%)	
TNM stage			
I/II	62 (47%)	69 (53%)	*0.029*^f^
III/IV	97 (36%)	176 (64%)	
Missing	1 (50%)	1 (50%)	

*TCGA*-*BLCA* The Cancer Genome Atlas bladder cancer cohort

Based on Pearson χ^2^ test, *p*-value is calculated using Fisher’s exact test; Italics values denote *p*-value < 0.05

^a^Papillary vs. non-papillary

^b^T1/T2 vs. T3/T4

^c^Negative vs. positive

^d^M0 vs. M1

^e^Negative vs. positive

^f^I/II vs. III/IV

### SELENBP1 expression inversely associates with DNA methylation in the promoter and gene body, but positively correlates with DNA methylation in 3′-UTR region

**Fig. 2:**
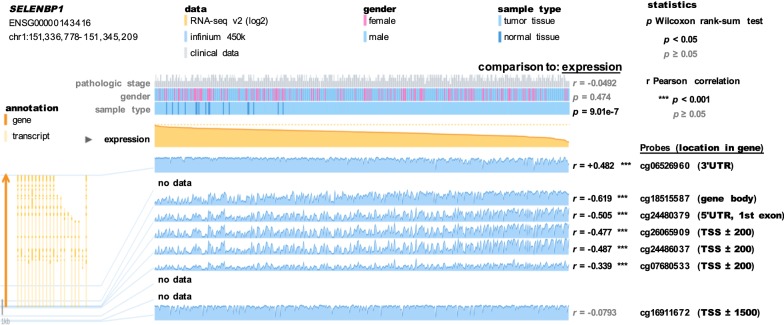
Visualization of the association between *SELENBP1* expression and DNA methylation in TCGA-BLCA cohort using the MEXPRESS tool. *SELENBP1* gene together with its transcripts is indicated in the left bottom panel. Next to the gene, blue line plots show the methylation data for the location of each probe (Infinium Human Methylation 450 microarray data; infinium 450 k), whose relative location in *SELENBP1* gene is also indicated in the far right panel. The yellow line plot displays the RNA-seq-derived expression data, and the samples are ordered by *SELENBP1* expression value. This view shows how *SELENBP1* expression and DNA methylation in different parts of SELENBP1 gene are correlated, which is confirmed by the Pearson correlation test. The correlation coefficients (r) are indicated in the right panel. The symbol (***) shows a significant correlation (*p *< 0.001). TSS, transcription start site; UTR, untranslated region

DNA methylation has been recognized as a major mechanism responsible for epigenetic silencing of SELENBP1 expression in esophageal and colorectal cancer [11, 42]. Therefore, we addressed this association between *SELENBP1* expression and DNA methylation in the bladder cancer by using the MEXPRESS tool (http://mexpress.be/), a user-friendly tool for the visualization and interpretation of DNA methylation in TCGA data [41]. As shown in Fig. 2, it was clear that normal samples cluster towards higher *SELENBP1* expression than tumor tissues (Wilcoxon rank-sum test, *p *= 9.01e−7). Moreover, there was a significant inverse association between *SELENBP1* expression and DNA methylation in its promoter region located close to the transcription start site (TSS) and the first exon (Pearson correlation coefficients ranging from − 0.339 to − 0.505; *p *< 0.001). Interestingly, we observed a more significant negative relationship between gene body methylation and *SELENBP1* expression with Pearson *r *= − 0.619 (*p *< 0.001). However, DNA methylation in its 3′ untranslated region (UTR) was positively associated with *SELENBP1* expression (Pearson *r *= + 0.482; *p *< 0.001). These observations highlight that those multiple genomic regions of DNA methylation may be differentially involved in modulating *SELENBP1* expression.

### Overexpression of SELENBP1 inhibits bladder cancer cell growth

**Fig. 3:**
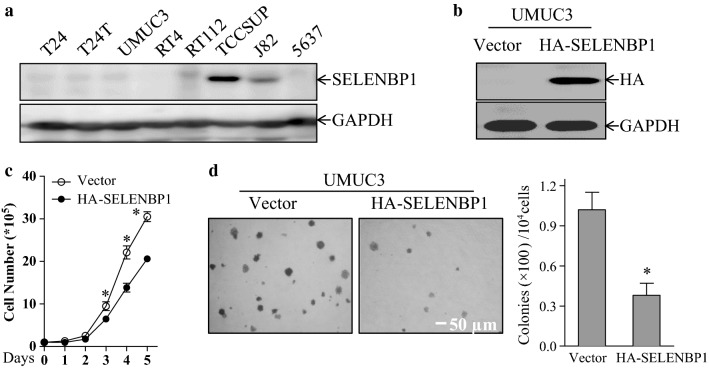
Overexpression of SELENBP1 inhibits bladder cancer cell growth. **a** Cell lysates of human bladder cancer cell lines were extracted and subjected to immunoblotting assay for determining the levels of SELENBP1 protein expression. GAPDH was used as a loading control. **b** UMUC3 cells were stably transfected with empty vector (Vector) or construct encoding HA-tagged SELENBP1 (HA-SELENBP1). Cell lysates of these stable transfectants were extracted and subjected to immunoblotting assay by using anti-HA antibody. GAPDH was used as a loading control. **c** The number of 1 × 10^4^ stable UMUC3 (Vector) and UMUC3 (HA-SELENBP1) cells per well was seeded in 6-well plates. The number of viable cells at the indicated time points was determined by trypan blue exclusion assay. The values were shown as mean ± standard deviation from at least three independent experiments. The asterisk (*) indicates a significant difference in comparison to that of control group (*p *< 0.05). **d** Anchorage-independent cell growth of UMUC3 cells stably transfected with empty vector or construct encoding HA-SELENBP1 was determined by soft agar assay. The colony formation was observed and captured under an inverted microscope (×40 magnification; *left panel*) and numbers of colonies were scored on day 15, and presented as colonies (×100)/10^4^ cells *(right panel)*. The values were shown as mean ± standard deviation from at least three independent experiments. The asterisk (*) indicates a significant difference in comparison to that of control transfectants (*p *< 0.05)

To determine the biological function of SELENPB1 in bladder cancer, we firstly employed immunoblotting assay to evaluate SELENBP1 expression in 8 of human bladder cancer cell lines, including T24, T24T, UMUC3, RT4, RT112, TCCSUP, J82 and 5637. As expected, the majority of bladder cancer cell lines exhibited undetectable SELENBP1 protein levels, except TCCSUP and J82 cell lines (Fig. 3a). Then, we established stable transfectants expressing HA-tagged SELENBP1 in UMUC3 cells (Fig. 3b). As shown in trypan blue exclusion assay (Fig. 3c), ectopic expression of SELENBP1 in UMUC3 cells led to a significant decrease in anchorage-dependent growth. Additionally, we also observed that overexpression of SELENBP1 markedly inhibited anchorage-independent growth of UMUC3 cells (Fig. 3d), further verifying the tumor-suppressive role for SELENBP1 in human bladder cancer cellular transformation.

### Induction of p21 protein is crucial for SELENBP1-mediated G_0_/G_1_ phase cell cycle arrest

**Fig. 4:**
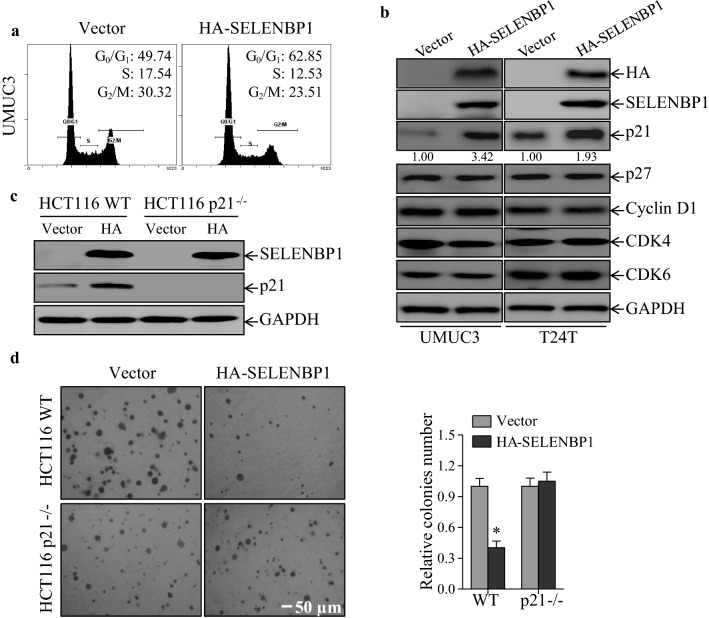
Induction of p21 protein is crucial for SELENBP1-mediated G_0_/G_1_ phase cell cycle arrest. **a** UMUC3 (Vector) and UMUC3 (HA-SELENBP1) cells were collected at day 4 as described in “Fig. 3c”, and subjected to flow cytometry assay following the staining of propidium iodide. **b** Cell lysates of stable transfectants UMUC3 (Vector), UMUC3 (HA-SELENBP1), T24T (Vector) and T24T (HA-SELENBP1) were extracted and subjected to immunoblotting assay for determining expression levels of G_0_/G_1_ phase arrest-associated regulatory proteins. GAPDH was used as a loading control. Densitometric quantification of p21 (relative to GAPDH) is shown under each blot. **c** HCT116 WT and HCT116 p21^−/−^ cells were transfected with empty vector or HA-SELENBP1, each along with pGIPZ, and stable transfectants were established under the selection of puromycin. Cell lysates of these stable transfectants were extracted and then subjected to immunoblotting assay with anti-SELENBP1, anti-p21 or anti-GAPDH antibodies. **d** Anchorage-independent cell growth of stable transfectants described in “**c**” was determined by soft agar assay. Representative images of colonies were visualized under an inverted microscope (×40 magnification; *left panel*) and relative numbers of colonies were calculated on day 12 *(right panel)*. The asterisk (*) indicates a significant difference in comparison to that of control transfectants (*p *< 0.05)

To elucidate the underlying mechanism responsible for its inhibitory role in cancer cell growth, the potential effect of SELENBP1 on cell cycle was determined by flow cytometry analysis. The results showed that overexpression of SELENBP1 induced a significant G_0_/G_1_ phase arrest in UMUC3 cells (Fig. 4a), suggesting that G_0_/G_1_ phase arrest induction might be associated with tumor suppressive role of SELENBP1. To further elucidate the crucial players that are involved in SELENBP1-mediated G_0_/G_1_ phase arrest, we then evaluated the regulatory effect of SELENBP1 on multiple cell cycle-associated proteins, including cyclin-dependent kinase inhibitor 1A (CDKN1A; also known as p21), cyclin-dependent kinase inhibitor 1B (CDKN1B or p27), cyclin D1, cyclin-dependent kinase 4 (CDK4) and CDK6. As shown in Fig. 4b, overexpression of SELENBP1 had no observable regulatory role on p27, Cyclin D1, CDK4 or CDK6, but did result in a dramatic induction of p21 protein in both of UMUC3 and T24T cells. This finding suggests that p21 protein is likely to be a crucial player in SELENBP1-mediated cancer cell growth inhibition. To verify this notion, we established stable transfectants expressing HA-tagged SELENBP1 protein in human colon cancer HCT116 wild-type cells (HCT116 WT) and p21 knockout cells (HCT116 p21^−/−^), in both of which endogenous protein levels of SELENBP1 were undetectable [30]. As expected, overexpression of SELENBP1 consistently stimulated p21 protein expression and further significantly inhibited anchorage-independent cell growth in HCT116 WT cells (Fig. 4c, d). However, SELENBP1 failed to modulate cancer cell growth in p21-deficiency cells (Fig. 4d), revealing that p21 protein serves as a crucial downstream mediator responsible for the growth-inhibitory role of SELENBP1 in cancer cells. Collectively, these results demonstrate that SELENBP1 specifically up-regulates p21 protein levels and induces G_0_/G_1_ phase arrest, thereby leading to attenuation of cancer cell growth.

### SELENBP1 transcriptionally stimulates p21 expression in a p53-independent manner

**Fig. 5:**
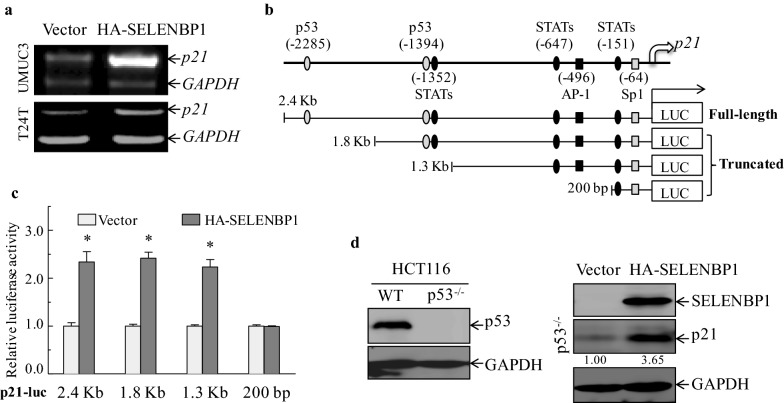
SELENBP1 transcriptionally stimulates p21 expression in a p53-independent manner. **a** Total RNA was isolated from indicated stable transfectants, and then subjected to RT-PCR assay for determining *p21* mRNA levels. The mRNA levels of *GAPDH* were used as a loading control. **b** The potential binding sites of multiple transcription factors in the *p21* promoter are shown in *upper panel*, and full-length (2.4 Kb), 1.8 Kb, 1.3 Kb and 200 bp of *p21* promoter-driven luciferase reporters are schematically shown in *lower panel*. **c** Stable UMUC3 (Vector) and UMUC3 (HA-SELENBP1) cells were transiently co-transfected with pRL-TK and *p21* promoter-driven luciferase reporters as described in “**b**”. Following 36 h of transient transfection, luciferase reporter activity was determined and normalized to that of corresponding UMUC3-Vector. The asterisk (*) indicates a significant difference in comparison to that of corresponding control transfectants (*p *< 0.05). **d** Cell lysates of HCT116 WT and HCT116 p53^−/−^ were subjected to immunoblotting assay for identification of p53 expression (*left panel*). Following 48 h of transient transfection with HA-SELENBP1 or empty vector, HCT116 p53^−/−^ cells were extracted and then subjected to immunoblotting assay with anti-SELENBP1, anti-p21 or anti-GAPDH antibodies (*right panel*). Densitometric quantification of p21 (relative to GAPDH) is shown

To investigate the mechanisms underlying SELENBP1-mediated induction of p21 protein, we first examined *p21* mRNA levels, and found that *p21* mRNA was markedly up-regulated in both UMUC3 and T24T stable transfectants expressing HA-SELENBP1 (Fig. 5a). To further verify whether SELENBP1-mediated induction of p21 expression occurs at transcriptional level, dual luciferase activities were evaluated in stable UMUC3 (Vector) and UMUC3 (HA-SELENBP1) transfectants using one full-length (2.4 Kb) and three differential truncated-length (1.8 Kb, 1.3 Kb and 200 bp) of human *p21* promoter-driven luciferase reporters (Fig. 5b). As indicated in Fig. 5c, overexpression of SELENBP1 significantly led to an approximately 2.3-fold increase in full-length (2.4 Kb), 1.8 Kb- or 1.3 Kb-length of *p21* promoter-driven luciferase activities, whereas this regulatory effect was not observed in 200 bp-length reporter assay. These findings reveals that SELENBP1 transcriptionally stimulates p21 expression through the potential responsive elements located within approximately ~ 1300 bp to ~ 200 bp region of the *p21* upstream promoter. It has been well established that tumor suppressor p53 is able to binding to the *p21* promoter and directly activate its expression [43]. However, we noticed that p53-responsive elements, located in − 1394 bp and − 2285 bp region of *p21* promoter [33, 43], are outside of identified SELENBP1-responsive region in *p21* promoter (Fig. 5b), leading us to the notion that SELENBP1-mediated p21expression is likely to occur through a p53-independent mechanism. To verify this notion, we re-introduced HA-tagged SELENBP1 in human colon cancer HCT116 p53 knockout cells (HCT116 p53^−/−^), and found that SELENBP1 still retained the ability to activate p21 expression even in the absence of p53 (Fig. 5d). This result indicates that p53 protein might not be required for SELENBP1-mediated regulation of p21 expression. Collectively, despite the well-established putative role for p53 in the transcriptional regulation of p21 expression, our results demonstrate that SELENBP1 triggers transcriptional induction of p21 expression through a p53-independent pathway.

### Overexpression of SELENBP1 results in the suppression of c-Jun and STAT1 phosphorylation

**Fig. 6:**
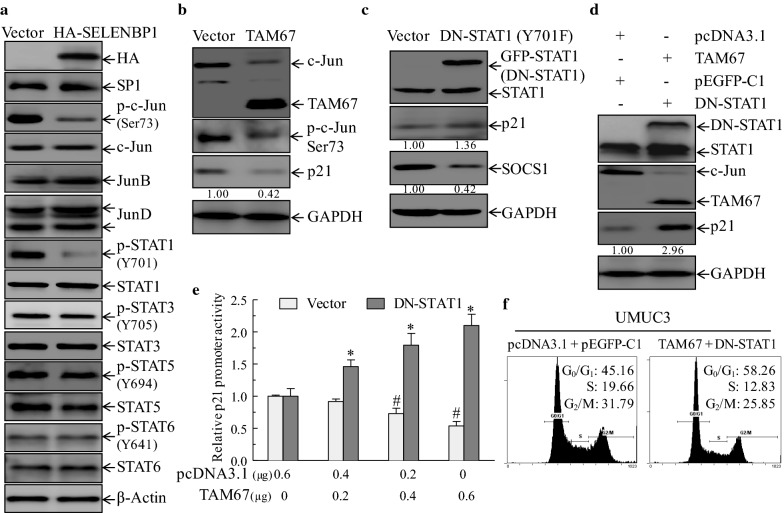
The restoration of SELENBP1 leads to the suppression of c-Jun and STAT1 phosphorylation, both of which are required for SELENBP1-mediated transcriptional induction of p21 protein. **a** Cell lysates of stable UMUC3 (Vector) and UMUC3 (HA-SELENBP1) cells were subjected to immunoblotting assay with indicated antibodies that might be involved in transcriptional regulation of p21 expression. **b** UMUC3 cells stably transfected with dominant-negative mutant form of c-Jun (TAM67) or pcDNA3.1(Vector) were extracted and then subjected to immunoblotting analysis with indicated antibodies. Densitometric quantification of p21 (relative to GAPDH) is shown. **c** UMUC3 cells were stably transfected with dominant-negative mutant STAT1 (DN-STAT1) or pEGFP-C1 construct (Vector), and were then extracted for immunoblotting assay with indicated antibodies. Densitometric quantification of p21 and SOCS1 (relative to GAPDH) is shown under each blot. **d** TAM67 (1 μg) and pcDNA3.1 (1 μg) plasmids were transiently transfected into stable UMUC3 (DN-STAT1) and UMUC3 (pEGFP-C1), respectively. Following 48 h of transient transfection, cells were collected and then subjected to immunoblotting assay. Densitometric quantification of p21 (relative to GAPDH) is shown. **e** Indicated amounts of empty vector (pcDNA3.1) and TAM67 was transiently co-transfected into stable UMUC3 (DN-STAT1) or UMUC3 (Vector) cells, together with 1.3 Kb of *p21* promoter-driven luciferase reporter and pRL-TK, as an internal control. Thirty-six hours post transfection, luciferase reporter activity was determined. Luciferase activities of UMUC3 (Vector) and UMUC3 (DN-STAT1) groups are normalized to those of corresponding control group that transfected with 0.6 μg of pcDNA3.1, respectively. The asterisk (*) indicates a significant difference as compared to UMUC3 (DN-STAT1) group transfected with 0.6 μg of pcDNA3.1 (*p *< 0.05). The symbol (#) indicates *p *< 0.05 when compared with UMUC3 (Vector) group transfected with 0.6 μg of pcDNA3.1. The values are shown as mean ± standard deviation from four independent experiments. **f** Following 48 h of transient transfection with indicated plasmids, UMUC3 cells were collected and then subjected to flow cytometry assay

In addition to p53 protein, bioinformatics analysis revealed that *p21* promoter region also contains multiple consensus binding sites for other transcriptional factors [44], including signal transductions and activators of transcription (STATs), activating protein-1 (AP-1) and specificity protein 1 (Sp1) (Fig. 5b). To identify whether these transcriptional factors are involved in SELENBP1-mediated transcriptional induction of p21, we used immunoblotting assay to evaluate the levels of transcription factors Sp1 and AP-1 subunits-JunB, JunD and c-Jun, as well as STAT family members, including STAT1, STAT3, STAT5 and STAT6. The results showed that both c-Jun phosphorylation at Ser73 and STAT1 phosphorylation at Tyr701 were significantly down-regulated in UMUC3 cells expressing HA-SELENBP1, whereas other evaluated transcription factors showed no observable changes (Fig. 6a). This result suggests that c-Jun and/or STAT1 may be involved in SELENBP1-mediated transcriptional induction of p21.

### Combined suppression of c-Jun and STAT1 is required for SELENBP1-mediated transcriptional induction of p21 protein

Next, to define the roles of c-Jun and STAT1 in the regulation of p21 protein, dominant-negative mutants of c-Jun (TAM67) [31] and STAT1 (DN-STAT1 Y701F) [32] were stably transfected into UMUC3 cells, respectively. The results showed that TAM67 efficiently down-regulated c-Jun phosphorylation at Ser73, but inhibited the expression of p21 protein (Fig. 6b), indicating that inhibition of c-Jun activity alone is not sufficient to mimic the effect of SELENBP1-mediated induction of p21 protein. Conversely, blockade of STAT1 activity alone by DN-STAT1 Y701F slightly increased the expression of p21 protein (Fig. 6c), though dominant-negative STAT1 resulted in an effective down-regulation of suppressor of cytokine signaling 1 (SOCS1), a negative regulator of JAK/STAT signaling [45]. These observations led us to hypothesize whether simultaneous blockade of c-Jun and STAT1 activity is required for the induction of p21 protein. To verify this notion, we introduced pcDNA3.1 control and TAM67 into stable UMUC3 (Vector) and UMUC3 (DN-STAT1 Y701F) transfectants, respectively. As shown in Fig. 6d, concurrent blockade of c-Jun and STAT1 activities resulted in a significant increase in the expression of p21 protein, revealing that suppression of both c-Jun and STAT1 activity is indispensable for SELENBP1-mediated induction of p21 protein. Consistent with the induction of p21 protein, the truncated-length (1.3 Kb) of human *p21* promoter-driven luciferase reporter assay revealed that *p21* promoter activity increased significantly following the introduction of increasing amounts of TAM67 into stable UMUC3 (DN-STAT1 Y701F) cells (Fig. 6e). Meanwhile, we noticed that inhibition of c-Jun phosphorylation by TAM67 alone attenuated *p21* promoter activity in stable UMUC3 (Vector) cells (Fig. 6e). This observation was, however, in agreement with TAM67-mediated inhibition of p21 protein (Fig. 6b), suggesting that c-Jun regulates p21 expression in a context-dependent manner, at least dependent on the STAT1 activity. More importantly, we found that simultaneous blockade of c-Jun and STAT1 activity induced a significant G_0_/G_1_ phase arrest in UMUC3 cells (Fig. 6f). These mechanistic insights further highlight the indispensable roles for both c-Jun and STAT1 in SELENBP1-mediated transcriptional induction of p21 protein and subsequent G_0_/G_1_ phase arrest. Taken together, our results demonstrate overexpression of SELENBP1 triggers transcriptional activation of p21 through concurrent suppression of c-Jun and STAT1 phosphorylation.

## Discussion

Reduced expression of selenium-binding protein 1 (SELENBP1), since first cloned in 1997, has been frequently observed in a variety of solid tumors, and is significantly related to unfavorable clinical outcome in multiple cancer types [23]. However, the evidence concerning the clinical relevance of SELENBP1 in human bladder cancer is lacking. In the present study, we are the first to show that the reduction of SELENBP1 expression is also a frequent event in human bladder cancer tissues (Fig. 1), but is more likely to occur in patients with non-papillary subtype and advanced TNM stage (Table 1). Furthermore, restoration of SELENBP1 expression results in a significant G_0_/G_1_ phase arrest and subsequent cell growth inhibition in human bladder cancer cells (Figs. 3 and 4). Collectively, we report here that SELENBP1 may serve as a promising prognostic biomarker in predicting poor clinical outcome among patients with bladder cancer. Nevertheless, how SELENBP1 is clinically incorporated into bladder cancer management together with other prognostic biomarkers merits further investigation.

Cytosine methylation is a DNA modification generally associated with epigenetic transcription silencing [46]. The publicly available TCGA-BLCA data, analyzed by the online MEXPRESS tool [41], indicates that multiple locations of *SELENBP1* gene are hyper-methylated in a majority of bladder cancer tumors (Fig. 2). In line with prior reports in esophageal and colorectal cancer [11, 42], DNA methylation in the immediate vicinity of the TSS is inversely associated with *SELENBP1* expression (Pearson correlation coefficients ranging from − 0.339 to − 0.505; *p *< 0.001). Intriguingly, this inverse association is much more significant between methylation in gene body and *SELENBP1* expression (Pearson *r *= − 0.619, *p *< 0.001), suggesting that DNA hypermethylation, especially in gene body, is likely to account for the reduction of *SELENBP1* expression in bladder cancer. However, there is a significant and positive relationship between DNA methylation in 3′-UTR region and *SELENBP1* expression (Pearson r = + 0.482; *p *< 0.001). This apparent paradox supports that DNA methylation in different genomic contexts might be differentially involved in the regulation of *SELENBP1* expression [47]. Recently, DNA methylation-based biomarker studies have predominantly been focused on the effects of hypermethylation of promoter in tumour-suppressor genes [48]. Therefore, our finding provides valuable information when developing SELENBP1 as a genomic methylation-based biomarker for tumor progression and prognostic outcome.

To date, the molecular functions of SELENBP1 in cancer are relatively poorly understood, although it has been consistently reported to attenuate aberrant cell growth and suppress cell migration in a variety of cancer cells [49–51]. We and others have previously shown that SELENBP1 has the ability to form a physical interaction with glutathione peroxidase 1 selenoprotein [17, 52], a well-characterized enzyme that detoxify hydrogen peroxide to nontoxic water [53]. These findings implies a novel role for SELENBP1 in modulating the cellular redox microenvironment, which is further supported by our recent report showing that SELENBP1 regulates the levels of extracellular reduced form of glutathione [30]. More recently, SELENBP1 has been recognized as a novel human methanethiol oxidase with an enzymatic role in sulfur metabolism [54]. Here, we establish a novel biochemical link between SELENBP1 and p21 protein, and further determine the indispensable role for p21 in SELENBP1-mediated growth inhibition in cancer cells (Fig. 4). However, whether glutathione or sulfur metabolism is involved in SELENBP1-mediated induction of p21 protein warrants further investigation.

The p21 protein, also known as p21^WAF1/CIP1^, is encoded by the *CDKN1A* gene mapped to chromosome 6p21.2, and belongs to the Cip/Kip family of CDK inhibitors [55]. A substantial number of studies have demonstrated p21 as a multifunctional, broad-acting protein that plays key roles in cell cycle regulation, cell migration and apoptosis [44]. Thus, p21 has been shown to be intricately regulated via p53-dependent and –independent pathways [56]. Here, we verify that SELENBP1 activates transcriptional induction of p21 through a p53-independent mechanism using p53-deficiency cancer cell model (Fig. 5). Given the high prevalence of TP53 mutation and/or lose of function across almost all of cancer types, our finding suggests that SELENBP1 probably retains the growth-inhibitory capacity even in p53-mutated cancer cells.

Independent of p53, a variety of transcription factors have been reported to transcriptionally activate p21 protein expression [56], including Sp1. However, there is no observable effect on this player following the restoration of SELENBP1 in bladder cancer cells (Fig. 6). By contrast, SELENBP1 specifically inhibits the phosphorylation of c-Jun and STAT1, belonging to AP-1 and STAT family, respectively. Our further analysis suggests that combined suppression of c-Jun and STAT1 activities may be essential for SELENBP1-mediated transcriptional induction of p21 protein. C-Jun has been previously shown to interact with STAT3 and co-operatively regulate the transcription of their target genes in bladder cancer [57]. Given that c-Jun responsive element is closely located at the potential STAT1 binding sites in the promoter of *p21*, it is intriguing to further determine whether there is a physical interaction between c-Jun and STAT1 in the context of SELENBP1-mediated p21 expression, and the results from which would improve our understanding of the tumor-suppressive role for SELENBP1 in cancer.

## Conclusion

In summary, our results have revealed that the reduction of SELENBP1 is a frequent event and significantly correlates with tumor progression as well as unfavorable prognosis among patients with bladder cancer. Further bioinformatics analysis suggests that DNA hypermethylation, especially in gene body, is likely to account for the reduction of *SELENBP1* expression in bladder cancer. More importantly, we have established a novel biochemical link between SELENBP1 and p21 protein, in which SELENBP1 activates transcriptional induction of p21 expression in a p53-independent mechanism. Taken together, our results provide clinical and molecular evidence for an improved understanding of the tumor-suppressive role of SELENBP1 in bladder cancer, suggesting that SELENBP1 could potentially be used as a prognostic biomarker as well as a therapeutic target in future cancer therapy. Therefore, how SELENBP1 is clinically incorporated into bladder cancer management merits further investigation.

## Data Availability

Data sharing is not applicable to this article as no datasets were generated or analyzed during the current study.

## Abbreviations

SELENBP1: selenium-binding protein 1
p21: cyclin-dependent kinase inhibitor 1A
p27: cyclin-dependent kinase inhibitor 1B
STAT1: signal transduction and activator of transcription 1
SOCS1: suppressor of cytokine signaling 1
AP-1: activating protein-1
Sp1: specificity protein 1
CDK4: cyclin-dependent kinase 4
CDK6: cyclin-dependent kinase 6
UTR: untranslated region
TSS: transcription start site
OS: overall survival
TCGA-BLCA: The Cancer Genome Atlas bladder cancer
